# 1′-Acetoxyeugenol Acetate Isolated from Thai Ginger Induces Apoptosis in Human Ovarian Cancer Cells by ROS Production via NADPH Oxidase

**DOI:** 10.3390/antiox11020293

**Published:** 2022-01-31

**Authors:** Ju-Yeon Choi, Na-Kyung Lee, Yi-Yue Wang, Joon-Pyo Hong, So Ri Son, Da-Hye Gu, Dae Sik Jang, Jung-Hye Choi

**Affiliations:** 1 Department of Biomedical and Pharmaceutical Sciences, Kyung Hee University, Seoul 02447, Korea; chlwndus001@khu.ac.kr (J.-Y.C.); bk0401@khu.ac.kr (N.-K.L.); wangyiyue@khu.ac.kr (Y.-Y.W.); ongjoon@naver.com (J.-P.H.); allosori@khu.ac.kr (S.R.S.); dsjang@khu.ac.kr (D.S.J.); 2 College of Pharmacy, Kyung Hee University, Seoul 02447, Korea; 2018102299@khu.ac.kr

**Keywords:** *Alpinia galanga*, 1′-acetoxyeugenol acetate, ovarian cancer, apoptosis, reactive oxygen species, NADPH oxidase

## Abstract

The rhizomes of *Alpinia galanga* (Thai ginger) have been used extensively as a spice in Southeast Asian and Arabian cuisines and reported to possess a wide range of biological properties, such as antioxidant, antimicrobial, and antibacterial. However, the specific molecular and cellular mechanisms underlying the anti-tumor effects induced by Thai ginger and its corresponding active compounds have been poorly characterized. We found that upon EtOH extraction, Thai ginger extract exhibits cytotoxic activity (IC_50_ < 10 μg/mL) and triggers cell death via caspase-dependent apoptosis in human ovarian cancer cells. Among the three major compounds isolated from the extract, 1′-acetoxyeugenol acetate (AEA) exhibited potent cytotoxic activity in human ovarian cancer cells, SKOV3 and A2780. AEA induced apoptotic cell death through the activation of caspases-3 and -9. Notably, AEA enhanced the intracellular levels of reactive oxygen species (ROS), and the application of an antioxidant markedly reversed AEA-induced apoptosis of ovarian cancer cells. The knockdown of p47phox, a subunit of NADPH oxidase, suppressed both the pro-apoptotic and ROS-inducing effects of AEA. Additionally, the activation of the mitogen-activated protein kinase (MAPK) pathway by AEA through ROS regulation was found to be involved in AEA-induced apoptosis. Altogether, these results suggest that AEA exhibits potent apoptosis-inducing activity through the activation of the intrinsic pathway via ROS-mediated MAPK signaling in human ovarian cancer cells.

## 1. Introduction

Ovarian cancer is the most fatal gynecological cancers around the world [1]. Since there are no unambiguous symptoms, patients are mostly diagnosed at stage 3 or 4, resulting in a 5-year survival rate of approximately 30% in the past 30 years [2]. Debulking surgery and chemotherapy with taxane and platinum agents remain the standard-of-care first-line treatments for ovarian cancer. Due to the severe adverse effects and drug resistance of taxane/platinum-based chemotherapy [3], a novel therapeutic agent that would help improve the quality of life and survival rate of patients with ovarian cancer is urgently needed.

*Alpinia galanga* (L.) Willd. is widely cultivated in subtropical and tropical regions and is especially abundant in Asian countries, such as China, Thailand, Indonesia, India, and the Philippines [4]. The rhizomes of *A. galanga*, commonly known as “Thai ginger”, have been used extensively as a spice in Southeast Asian and Arabian cuisines. For example, the rhizomes are common ingredients in Thai curries and soups, such as tom kha kai, and used as fresh chunks, sliced, crushed, and mixed into a curry paste, or used to create a spicy taste [5]. Thai ginger has been used to treat eczema, diarrhea, abdominal pain, and cholera as part of traditional medicine [6,7]. Recent experimental studies have demonstrated that ginger extracts have various biological properties, such as anti-inflammatory, antimicrobial, antioxidant, antibacterial, and anti-tumor [8,9,10,11,12]. 1′-Acetoxychavicol acetate (ACA), one of the major constituents of Thai ginger, has been intensively studied as a major active compound for the bioactivity of ginger. To date, numerous pharmacological properties of ACA have been reported, including anti-tumor, anti-obesity, anti-allergy, anti-microbial, anti-diabetic, gastroprotective, and anti-inflammatory [13,14,15,16]. In particular, the inhibitory effect of ACA on carcinogenesis has been demonstrated in several animal models of various tumor types, including colon, liver, esophageal, skin, and prostate cancers [17,18,19,20,21]. Moreover, ACA has been shown to induce apoptosis and cell cycle arrest and inhibit angiogenesis and invasion in several cancer cell lines [22,23]. However, little is known regarding the effects of 1′-acetoxyeugenol acetate (AEA), another active compound in *A. galanga*, on human cancer. Moreover, there is no report regarding the effects of Thai ginger and its major constituents, including ACA and AEA, on human ovarian cancer. Therefore, in this study, we investigated the effect of Thai ginger extracts and the corresponding isolated compounds on human ovarian cancer cells and the underlying molecular mechanisms.

## 2. Materials and Methods

### 2.1. Sample Preapration

The dried rhizomes of *A. galanga* (Zingiberaceae) used in the study were provided by GMP BIO Co., LTD. (Seoul, South Korea) in June 2018. A specimen (ALGA-2018) has been stored in the Laboratory of Natural Product Medicine, College of Pharmacy, Kyung Hee University. The origin of *A. galanga* rhizome was confirmed by Dae Sik Jang. The dried and powdered rhizomes of *A. galanga* (100 g) were extracted twice with hot water (1 L) at 100 °C in a water bath for 2 h. To give a water extract (21 g), the solvent was evaporated at 45 °C in vacuo. As for the EtOH extract, the dried and powdered rhizomes (1.0 kg) were extracted with 95% EtOH (10 L) at room temperature three times, each for 24 h. The EtOH extract (47.78 g) was fractionated using silica gel column chromatography (CC) (230–400 mesh, *ϕ* 6.6 × 44.0 cm, *n*-hexane-EtOAc = 8.5:1.5 to 5:5, *v*/*v*) to obtain 24 fractions (E1~E24). Fraction E9 (6.41 g) was separated into six subfractions (E9-1~E9-6) using Sephadex LH-20 CC (*ϕ* 3.6 × 65.0 cm), with CH_2_Cl_2_-MeOH (5:5, *v*/*v*). 1′-Acetoxychavicol acetate (3.98 g) was purified from subfractions E9-2 and E9-3 using reversed-phase CC (YMC gel 75 μm, *ϕ* 3.8 × 30.0 cm). Eugenyl acetate (14.6 mg) was separated by a flash chromatographic system with a Redi Sep-C_18_ cartridge (43 g, acetonitrile-H_2_O = 3.5:6.5 to 7:3, *v*/*v*) from fraction E6 (175.3 mg). Fraction E13 (453.8 mg) was chromatographed over silica gel CC (230–400 mesh, *ϕ* 2.9 × 32.0 cm, *n*-hexane-CH_2_Cl_2_-MeOH = 8.5:1:1 to 7:2:2, *v*/*v*/*v*) to afford 1′-acetoxyeugenol acetate (36.8 mg). The structure of AEA was identified by comparing previously reported spectroscopic data [24] (Supplementary Figure S1). The purity of AEA was determined as >95% by using UPLC (ultra-performance liquid chromatography) and NMR (nuclear magnetic resonance) experiments (Supplementary Figures S2–S4). The extracts and compounds were stored in a −20 °C freezer before the experiment.

*[1′S]-1′-Acetoxyeugenol acetate (AEA)* Colorless oil; [α]_D_^21^: −16.6° (*c* 0.21, EtOH); ^1^H-NMR (500 MHz, chloroform-*d*) *δ*_H_ 7.01 (1H, d, *J* = 8.5 Hz, H-2), 6.94 (2H, m, H-3,6), 6.25 (1H, dt, *J* = 6.0, 1.0 Hz, H-1′), 5.99 (1H, ddd, *J* = 17.0, 10.5, 6.0 Hz, H-2′), 5.31 (1H, dt, *J* = 17.0, 1.0 Hz, H-3′a), 5.26 (1H, dt, *J* = 10.5, 1.0 Hz, H-3′b), 3.83 (3H, s, OCH_3_), 2.31 (3H, s, OCOCH_3_), 2.12 (3H, s, OCOCH_3_); ^13^C-NMR (125 MHz, chloroform-*d*) *δ*_c_ 170.1 (OCOCH_3_), 169.2 (OCOCH_3_), 151.2 (C-2), 139.7 (C-1), 137.9 (C-4), 136.1 (C-2′), 123.0 (C-6), 119.8 (C-5), 117.2 (C-3′), 111.6 (C-3), 75.9 (C-1′), 56.1 (OCH_3_), 21.4 (OCOCH_3_), 20.9 (OCOCH_3_).

### 2.2. Cell Culture

Human ovarian cancer cell lines (SKOV3 and A2780) and a human normal kidney cell line (HEK293) were originally obtained from the American Type Culture Collection (ATCC; Manassas, VA, USA). Roswell Park Memorial Institute (RPMI) 1640, fetal bovine serum (FBS), streptomycin sulfate, and penicillin were procured from Life Technologies Inc. (Grand Island, NY, USA). The ovarian cancer cells were cultured in RPMI 1640 medium containing 5% FBS, streptomycin sulfate (100 μg/mL), and penicillin (100 U/mL) in a 5% CO_2_ at 37 °C. Caspase inhibitors z-VAD-fmk, z-IETD-fmk, and z-LEHD-fmk were purchased from Calbiochem (Bad Soden, Germany). *N*-Acetyl-l-cysteine (NAC) and all inhibitors for MAPKs were acquired from Sigma Chemical (St. Louis, MO, USA).

### 2.3. Cell Viability Assay

SKOV3 and A2780 cells were seeded at a density of 1.0 × 10^5^ cells/mL in a 96-well plate containing 50 μL of RPMI medium in each well and incubated for 24 h. Various concentrations of extracts and compounds dissolved in dimethyl sulfoxide (DMSO) were mixed with RPMI 1640 medium and added into cells in each well. The final concentration of DMSO in the medium did not exceed 0.1%. Following 24 h or 48 h incubation, 50 μL of MTT (3-(4,5-Dimethylthiazol-2-yl)-2,5-diphenyl tetrazolium bromide) solution was added into each well to achieve a final concentration of 0.5 mg/mL and then incubated for an additional 4 h [25]. MTT was procured from Molecular Probes Inc. (Eugene, OR, USA). After discard of the medium, the intracellular formazan crystal was dissolved in DMSO (50 μL). A microplate spectrophotometer (SpectraMax; Molecular Devices, Sunnyvale, CA, USA) was used to measure the optical density at 540 nm.

### 2.4. Annexin V and PI Double Staining for Apoptosis Analysis

Annexin V-fluorescein isothiocyanate (FITC) detection kit was purchased from BioBud (Seoul, South Korea). SKOV3 and A2780 cells were seeded at a density of 1.0 × 10^5^ cells/mL and incubated for 24 h. After treatment with EtOH extract (5, 10, and 20 µg/mL) or AEA (2.5, 5, and 10 µM for A2780 and 6, 12, and 24 µM for SKOV3) for 24 h, the cells were harvested, washed twice with ice-cold phosphate-buffered saline (PBS), and mixed with 500 μL of binding buffer (2.5 mM CaCl_2_, 10 mM HEPES, 140 mM NaCl, PH 7.4). In total, 1.25 μL of FITC-conjugated Annexin V was added into the suspended cells, and the mixture was incubated for 15 min. Then, 10 μL of PI (50 mg/mL) was added and incubated for 5 min in the dark place. The fluorescence of the cell mixtures was assessed by using Guava^®^ easyCyte flow cytometry (EMD Milipore, Bilerica, MA, USA).

### 2.5. Western Blotting Analysis

A2780 cells were treated with AEA (2.5, 5, or 10 μM) in the presence or absence of NAC (5 mM) for 24 h. Following the treatment, the cells were harvested and washed three times with ice-cold PBS. In order to obtain the total cellular proteins, the cells were lysed with a protein lysis buffer (Intron Biotechnology, Seoul, South Korea) according to the manufacturer’s instructions. Bradford assay was used for the protein determination. The protein mixture was denatured using 5× SDS (sodium dodecyl sulfate) sample buffer and 5 min boiling at 95 °C. SDS-PAGE (polyacrylamide gel electrophoresis) was performed using the denatured samples. After SDS-PAGE, separated proteins were transferred to polyvinylidene difluoride membranes (EMD Milipore), which were blocked with 5% skim milk for 1 h afterward. After discarding the blocking solution, primary antibodies (Supplementary Table S1) were added into the membranes in 1% skim milk for incubation overnight at 4 °C. After a subsequent washing three times with TBS-T (Tris-buffered saline containing Tween-20), the membrane was incubated with a secondary antibody at room temperature for 2 h. Secondary antibodies were procured from The Jackson Laboratory (West Grove, PA, USA). TBS was purchased from Boster Biological Technology Ltd. (Wuhan, China). To visualize the immunoreactive bands, an enhanced chemiluminescence (ECL) kit (EMD Millipore) and Image Quant Las-4000 (GE Healthcare Life Science, WI, USA) were used. The relative intensity of protein bands was assessed by Image J software (NIH, Bethesda, MD, USA).

### 2.6. Determination of Intracellular Reactive Oxygen Species

Dichlorofluorescein diacetate (DCFH-DA) (Santa Cruz Biotechnology, Santa Cruz, CA, USA) was used to measure the intracellular levels of ROS in human ovarian cancer cells. SKOV3 and A2780 cells were seeded at a density of 1.0 × 10^5^ cells/mL and treated with AEA (5 µM) for the desired time intervals. The cells were stained with DCFH-DA (20 µM) at 37 °C for 30 min. The fluorescent intensity was analyzed by Guava^®^ easyCyte flow cytometry.

### 2.7. Gene Knockdown Using siRNA

Small interfering RNAs (siRNAs) for p47phox (NM_000265) were purchased from Dharmacon (Lafayette, CO, USA), while non-specific siRNA was obtained from Bioneer technology (Daejon, South Korea). The cells were plated in 6-well plates and grew for 24 h before transfection. After cells were cultured to 70–80 % confluence, the cells were incubated with a transfection mixture and allowed to recover for an additional 24 h before the experiment according to the manufacturer’s instructions. Each transfection mixture contained siRNA at a final concentration of 20 nM and lipofectamine^®^ RNAiMAX transfection reagent (Invitrogen, Carlsbad, CA, USA) in serum-free Opti-MEM. Real-time RT-PCR was used to confirm the downregulation of p47phox. Amplification of cDNA was carried out according to the manufacturer’s instruction using an SYBR Premix Ex Taq™ Kit (TAKARA, Kyoto, Japan). Duplicate measurements were used to calculate the mean cycle threshold (Ct) of p47phox, and Ct values were normalized with the mean Ct of β-actin (NM_001101), a control gene. The primers used in this study were as follows: for p47phox sense primer, 5′-GTCAGATGAAAGCAAAGCGA-3′, for anti-sense primer, 5′-CATAGTTGGGCTCAGGGTCT-3′, for β-actin sense primer, 5′-CAAACATGATCTG GGTCATC-3′, for anti-sense primer, 5′-GCTCGTCGTCGACAACGGCT-3′.

### 2.8. Statistical Analysis 

The data are shown as the mean ± SD. Student’s *t*-test and one-way analysis of variance were used to determine statistically significant differences. *p*-value < 0.05 was considered to be statistically significant.

## 3. Results

### 3.1. Cytotoxicity Effect of EtOH Extract and Isolated Compounds of Thai Ginger on Human Ovarian Cancer Cells

**Figure 1:**
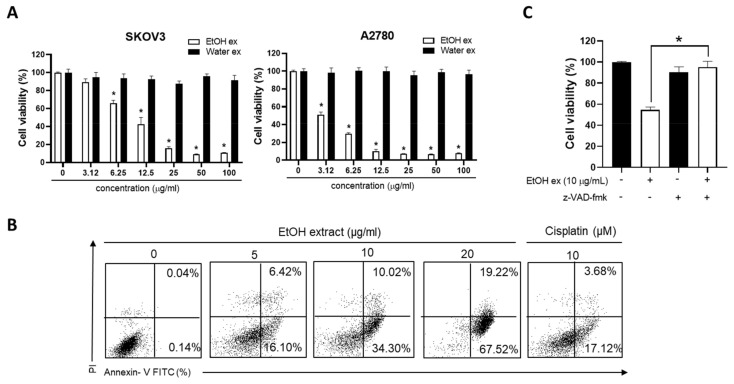
Effects of EtOH extract on cell viability and caspase-dependent apoptosis in human ovarian cancer cells. (**A**) SKOV3 and A2780 cells were treated with EtOH and water extracts for 48 h. MTT assay was performed for the cell viability. * *p* < 0.05 compared with control (**B**) A2780 cells were incubated with 5, 10, and 20 µg/mL of EtOH extract and cisplatin (10 µM) for 24 h. Annexin-V-PI double staining assay was performed. (**C**) After pretreatment with z-VAD fmk (50 µM), a broad caspase inhibitor, for 2 h, A2780 cells were treated with the EtOH extract (10 µg/mL) for 24 h. MTT assay was used to analyze cell viability. Data from one representative experiment of three independent experiments are shown * *p* < 0.05.

The effect of water and EtOH extracts of Thai ginger on the viability of human ovarian cancer cells was assessed. The EtOH extract of Thai ginger exhibited potent cytotoxic activity in two ovarian cancer cell lines, SKOV3 and A2780, with IC_50_ values below 10 µg/mL; however, the water extract of Thai ginger did not exert an effect on the cell viability (Figure 1A). Annexin V-PI double staining revealed that the EtOH extract induced a significant increase in the number of apoptotic cells (Figure 1B). Additionally, z-VAD fmk, a broad caspase inhibitor, significantly suppressed cell death induced by the EtOH extract of Thai ginger (Figure 1C). These results suggest that the EtOH extract of Thai ginger induces caspase-dependent apoptosis in human ovarian cancer cells. 

To identify the compounds from the EtOH extract of Thai ginger with cytotoxic activity against human ovarian cancer cells, we examined the effect of three major compounds, including 1′-acetoxychavicol acetate (ACA), eugenyl acetate, and 1′-acetoxyeugenol acetate (AEA) (Table 1). ACA and AEA, but not eugenyl acetate, exhibited a significant cytotoxic effect against both SKOV3 and A2780 cells, with IC_50_ values in the range of 10.01–14.13 μM, indicating that 7-acetoxy groups in ACA and AEA are important to exert cytotoxicity. Cisplatin, a positive control, showed significant cytotoxicity on human normal kidney HEK293 cells (IC_50_ value of 6.56 μM) as well as SKOV3 and A2780 cells (IC_50_ values of 21.13 μM and 19.00 μM, respectively). This observation is consistent with that nephrotoxicity is a well-known side effect of cisplatin in ovarian cancer treatment. Notably, ACA and AEA showed weak cytotoxicity against HEK293 cells compared with cisplatin.

To the best of our knowledge, this is the first report demonstrating the cytotoxic activity of Thai ginger extract, ACA, and AEA against human ovarian cancer cells. Although the anti-tumor activity and underlying molecular mechanism of ACA have been extensively studied in various types of cancer cells, the anti-tumor activity of AEA has been poorly characterized. Therefore, we further examined the molecular mechanism underlying the inhibitory effect of AEA on ovarian cancer cell viability.

### 3.2. AEA Leads to Cell Death via Caspase-Dependent Apoptosis in Human Ovarian Cancer Cells

**Figure 2:**
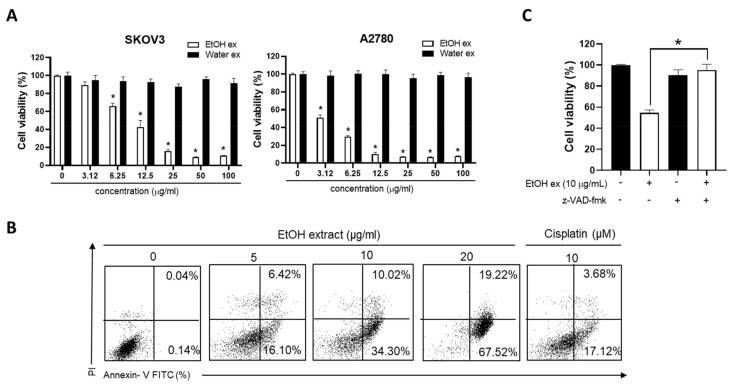
Effect of AEA on apoptosis in human ovarian cancer cells (**A**,**B**) SKOV3 (**A**) and A2780 (**B**) cells were incubated with AEA for 24 h. Apoptotic cell death analysis was performed by flow cytometry after Annexin V/PI staining double staining. Data from one representative experiment of three independent experiments are shown. * *p* < 0.05 compared with control.

**Figure 3:**
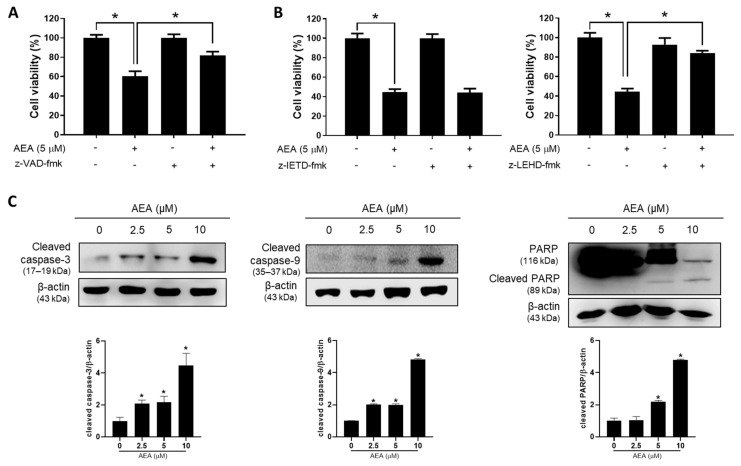
Involvement of caspases in AEA-induced apoptosis in human ovarian cancer cells. (**A**) A2780 cells were treated with AEA (5 µM) for 24 h following a 2 h pretreatment with a broad caspase inhibitor z-VAD-fmk (50 μM). Cell viability was determined using an MTT assay. (**B**) A2780 cells were pretreated with z-IETD-fmk (a caspase-8 inhibitor; 50 µM) and z-LEHD-fmk (caspase-9 inhibitor; 75 µM) for 2 h, and then treated with AEA (5 µM) for 24 h. Cell viability was measured using an MTT assay. (**C**) A2780 cells were treated with the indicated concentration of AEA (2.5, 5, and 10 µM) for 24 h. Cleaved caspase-3 and caspase-9 and PARP levels were analyzed by Western blotting. Results are representative of three independent experiments. * *p <* 0.05.

AEA enhanced the number of annexin V-positive apoptotic cells in a dose-dependent manner, as observed in the flow cytometry analyses of both SKOV3 and A2780 cells (Figure 2A,B), suggesting that apoptosis was involved in inhibiting the cell growth mediated by AEA. In the majority of cases, caspases act as mediators to activate the apoptosis pathway [26]. Z-VAD-fmk (a broad caspase inhibitor) significantly reversed AEA-induced cell death (Figure 3A), indicating that AEA induced caspase-dependent apoptosis. Therefore, z-IETD-fmk (caspase-8 inhibitor) and z-LEHD-fmk (caspase-9 inhibitor) were used to investigate whether AEA-induced caspase-dependent apoptosis is related to intrinsic or extrinsic pathways. z-LEHD-fmk, but not z-IETD-fmk, significantly affected AEA-induced cell death (Figure 3B). Consistent with the study on inhibitory effects, AEA treatment significantly enhanced the levels of cleaved forms of caspase-3 and -9 and PARP, a well-known caspase-3 substrate (Figure 3C). These results suggested that AEA induces intrinsic caspase-dependent apoptosis in human ovarian cancer cells.

### 3.3. ROS Production Is Involved in AEA-Induced Apoptosis through NADPH Oxidase Activation

**Figure 4:**
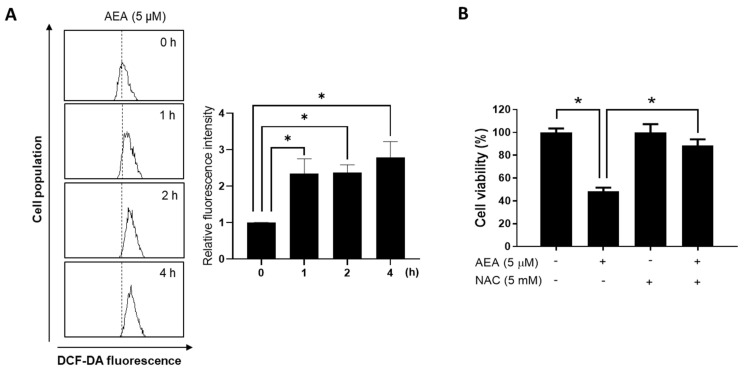
Involvement of ROS production in AEA-induced cell death in human ovarian cancer cells. (**A**) A2780 cells were treated with AEA (5 µM) for the indicated times (1, 2, and 4 h). After staining with DCF-DA (2′,7′-dichlorofluorescin diacetate) according to the manufacturer’s protocol, the cells were subjected to flow cytometry analysis. (**B**) After pretreatment with *N*-Acetyl-l-cysteine (NAC, 5 mM) for 30 min, the cells were treated with AEA (5 µM) for 24 h. Cell viability was measured using an MTT assay. Results are representative of three independent experiments. * *p <* 0.05.

**Figure 5:**
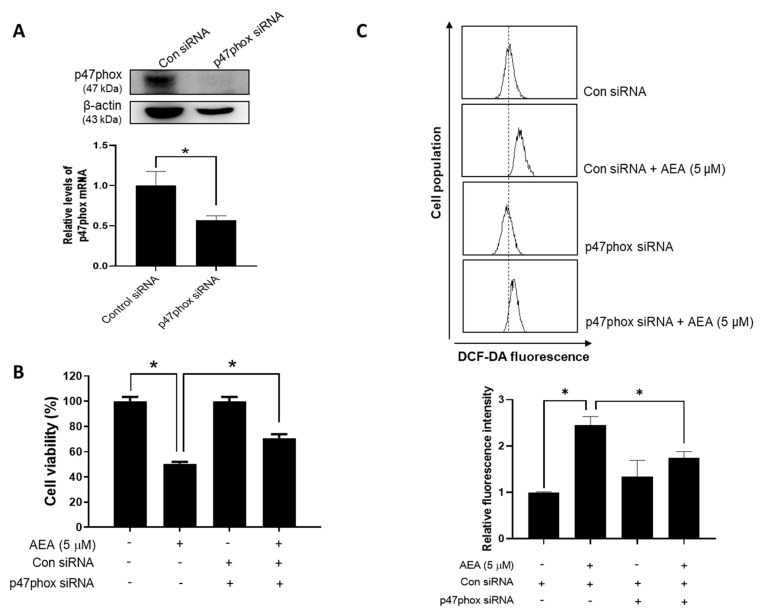
Involvement of the NADPH oxidase activation in AEA-induced apoptosis in human ovarian cancer cells. (**A**) A2780 cells were transfected with control siRNA or p47phox siRNA (20 nM). The levels of p47phox mRNA and protein were determined by real-time RT-PCR and Western blotting, respectively. (**B**) After the transfection with control or p47phox siRNA for 24 h, the cells were treated with AEA (5 µM) for 24 h. Cell viability was measured using an MTT assay. (**C**) A2780 cells were stained with DCF-DA and then analyzed using flow cytometry. Data from one representative experiment of three independent experiments are shown. * *p <* 0.05.

An increase in the intracellular reactive oxygen species (ROS) has been shown to induce apoptotic cell death in cancer cells [27]. Therefore, we evaluated the effect of AEA on the intracellular ROS levels in human ovarian cancer cells. AEA treatment led to an increase in the fluorescence intensity of DCF-DA in A2780 cells (Figure 4A), suggesting enhanced levels of ROS. Additionally, the antioxidant NAC markedly inhibited the effect of AEA on cell viability (Figure 4B). These data suggest that AEA induces ovarian cancer cell death via intracellular ROS production. We further investigated whether NADPH oxidase (NOX), a transmembrane enzyme that induces ROS production via NADPH oxidation, is associated with AEA-induced apoptosis using siRNA targeting p47phox, a subunit of NOX. The knockdown of p47phox in A2780 cells (Figure 5A) significantly reduced the inhibitory effect of AEA on cell viability (Figure 5B). Additionally, the increase in intracellular ROS levels by AEA was alleviated through p47phox knockdown (Figure 5C). These results indicate that NOX activation is involved in AEA-induced ROS generation and apoptosis in human ovarian cancer cells.

### 3.4. MAPKs Activation Is Involved in AEA-Induced Apoptosis

**Figure 6:**
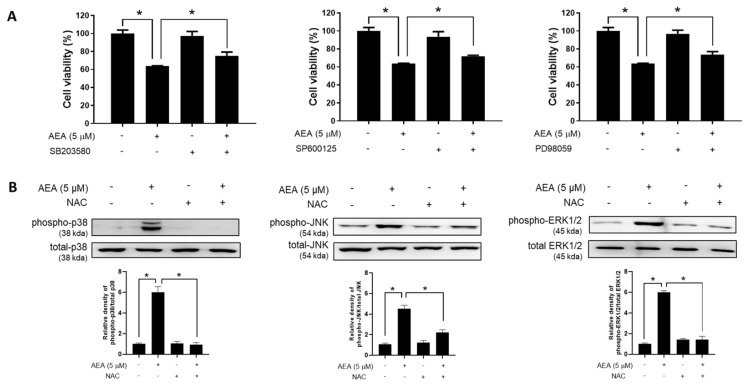
Involvement of MAPK pathways in AEA-induced cell death in human ovarian cancer cells (**A**) A2780 cells were pretreated with a p38 inhibitor SB203580 (1 µM), JNK inhibitor SP600125 (1 µM), or ERK inhibitor PD98059 (0.5 µM) for 30 min, and then treated with AEA (5 µM) for 24 h. MTT assay was carried out to determine cell viability. (**B**) After pretreatment with *N*-Acetyl-l-cysteine (NAC, 5 mM) for 30 min, and then treated with AEA (5 µM) for 24 h. The total and phosphorylated levels of p38, JNK, and ERK1/2 were analyzed by Western blotting. Band images are representatives of three independent experiments. * *p <* 0.05.

The mitogen-activated protein kinases (MAPKs) include ERK1/2, p38, and JNK subfamilies, which are key regulators of cell growth and apoptosis in cancer cells [28]. To investigate whether the activation of p38, JNK, and ERK1/2 is required for AEA-induced cell death, specific inhibitors of these signaling pathways were used. Here, we found that SB203580 (p38 inhibitor), SP600125 (JNK inhibitor), and PD98059 (ERK1/2 inhibitor) significantly dampened the growth inhibitory effect induced by AEA (Figure 6A), suggesting that the MAPKs are involved in the AEA-induced cell death. Considering that AEA induces ovarian cancer cell death via intracellular ROS production and ROS is known to activate the MAPKs, we further examined whether ROS mediate the activation of p38, JNK, and ERK1/2 stimulated by AEA. Pretreatment with antioxidant NAC markedly attenuated AEA-induced activation of p38, JNK, and ERK1/2 in A2780 cells (Figure 6B). These data suggest that an ROS-regulated MAPK signaling pathway is required for AEA-induced apoptosis in human ovarian cancer cells.

## 4. Discussion

Recently, various types of Thai ginger extracts have been reported to exhibit a wide range of biological activities [29,30]. For instance, in LPS-activated mouse peritoneal macrophages, acetone extract showed a suppressive effect on NO production [31]. Its MeOH extract showed anti-estrogenic activity in ovariectomized C3H albino mice [32]. As for the anti-tumor activities, the EtOH extracts are largely reported to exhibit apoptosis-inducing and anti-proliferative effects in many types of cancers, including breast and prostate cancer cells [33,34]. Interestingly, the water extract, similar to the mode of dietary consumption of Thai ginger, also showed growth inhibitory effects on gastric and lung cancer cells [35,36]. In this regard, we examined the effect of both EtOH and water extracts of Thai ginger on human ovarian cancer cells and found that EtOH extract exhibits a potent cytotoxic activity with an approximate IC_50_ value below 10 µg/mL. In contrast, water extract did not show an effect on the cell viability in ovarian cancer as well as other cancer cells (Supplementary Figure S5). Moreover, the cytotoxic effects of the EtOH extract of Thai ginger were associated with caspase-dependent apoptosis. Notably, this is the first study to demonstrate its anti-tumor activity against ovarian cancer cells. Moreover, it was more cytotoxic against ovarian cancer cells than other cancer cells, including breast cancer cells (IC_50_ = 400.0 ± 11.7 µg/mL) [33].

The phytochemical studies have revealed the abundance of phenylpropanoids, terpenes, and alkaloids in *A. galanga* [7,30]. Various biological activities of *A. galanga* have been attributed to its phenylpropanoid components, which are aromatic compounds with n-propyl groups [31,37]. In this study, ACA and AEA have been demonstrated to exhibit potent cytotoxicity against ovarian cancer cells, and both are phenylpropanoid compounds. This is the first report showing the cytotoxic and apoptotic effects of ACA and AEA against ovarian cancer. Moreover, the anti-tumor properties of ACA against several other cancers and its molecular mechanism of action have been intensively studied, as also reviewed in a previous study [22]. For example, Ichikawa et al. demonstrated that ACA inhibits invasion and induces apoptosis via inhibition of the NF-κB signaling pathway in several cancer cell types, including lung, kidney, and breast cancer [38]. Ito et al. reported that ACA induces apoptosis through upregulation of both TRAIL/Apo2L and TRAIL receptor death receptor 5 in multiple myeloma cells [39]. More recently, multiple studies have suggested that ACA regulates some microRNA (miRNA) expression in head and neck and cervical cancer cells [40,41,42]. However, only a few reports have shown the effect of AEA on breast cancer cells. Hemi-synthesized AEA has been shown to induce apoptosis and inhibit the migration of MDA-MD-231 cells [43]. AEA isolated from *A. chonchigera* was shown to induce apoptosis and inhibit the NF-κB pathway in MCF7 breast cancer cells [44]. However, the direct involvement of the NF-κB pathway in AEA-induced apoptosis has not been experimentally demonstrated yet. In this study, we revealed that AEA induced apoptosis by activating the intrinsic pathway through the regulation of the levels of intracellular ROS in human ovarian cancer cells. Notably, AEA was more cytotoxic against ovarian (IC_50_ = 14.13 µM for SKOV3 and IC_50_ = 10.01 µM for A2780, shown in Table 1) than breast (IC_50_ = 29.63 µM for MCF7, as shown in Supplementary Table S2) cancer cells. 

Except for anti-tumor activity, there are only a few reports on other biological properties of AEA isolated from various *Alpinia* species. AEA exhibits antimicrobial activity against pathogenic bacteria, diminishes stress induced by the HPA axis, and regulates neuroendocrine and neuroimmune functions to improve cognition [45]. However, the mechanism of action of AEA is poorly understood. Cellular signaling regulated by natural compounds varies depending on the compound [46,47]. Among various biological activities, programmed cell death and apoptosis are mostly involved in cell growth and regulation of the immune system, and the apoptosis-inducing effect of many natural products is a key factor for their anti-tumor activity [48]. Therefore, apoptosis is a promising therapeutic target for establishing cancer treatment. Apoptosis is activated via two well-characterized pathways in mammalian cells [49]. The first is the extrinsic pathway that involves the activation of caspase-8 and -3 through triggering a death receptor, such as TNF, expressed on the cell surface [50]. The second is an intrinsic pathway that is mediated by molecules released from the mitochondria, thereby activating caspase-9 and -3 [48]. In this study, we demonstrated that AEA activates the caspase-dependent intrinsic apoptosis pathway in human ovarian cancer cells. 

Certain natural compounds have been demonstrated to induce the intrinsic apoptosis pathway via ROS production in human cancer cells [51]. Interestingly, well-studied and closely related ACA and sodium butyrate have synergistically induced apoptosis via an increase in the levels of intracellular ROS in liver cancer cells [52]. Similarly, AEA treatment increased the intracellular levels of ROS and antioxidant NAC treatment inhibited AEA-induced caspase 9-dependent cell death, which suggests that the increase in ROS levels induced by AEA are mediated via the intrinsic apoptosis pathway in human ovarian cancer cells. ROS are specifically produced as by-products of the respiratory chain via mitochondria and are mainly activated by oxidases, such as NOX, arachidonic acid oxygenase, and xanthine oxidase [53,54]. Among these enzymes, NOXs are transmembrane proteins that produce ROS via NADPH oxidation. Additionally, NOXs have been shown to play important roles in tumorigenesis and cell proliferation [55]. We found that specific inhibition of p47phox, a subunit of NOXs, alleviated AEA-induced cell death and ROS production in A2780 cells. These data indicate that the activation of NOXs is involved in AEA-induced apoptosis via ROS accumulation in ovarian cancer cells. Further studies are required to elucidate the exact molecular mechanisms by which AEA induces ROS production. The upregulation of ROS levels has been shown to inhibit cell growth and induce apoptosis in human ovarian cancer through activating both the caspase and MAPK pathways [56]. MAPKs play a vital role in various cellular activities, including apoptosis, differentiation, and cell proliferation [57]. We found that MAPK inhibitors partially but significantly suppressed the apoptotic activity mediated by AEA in ovarian cancer cells. Additionally, NAC treatment decreased the AEA-induced activation of JNK, ERK1/2, and p38. These results indicate that ROS-mediated apoptosis induced by AEA involves MAPK signaling pathways in human ovarian cancer cells. 

This is the first study to demonstrate the anti-tumor activity of Thai ginger extract and AEA against human ovarian cancer cells. We demonstrated that AEA stimulates ROS-mediated caspase-dependent apoptosis of ovarian cancer via MAPK signaling (Supplementary Figure S6). These findings expand our knowledge of the health benefits of Thai ginger and AEA.

## Figures and Tables

**Table 1:** Cytotoxic activity of compounds isolated from the EtOH extract of Thai ginger in human ovarian cancer cells (SKOV3 and A2780) and normal kidney cells (HEK293).

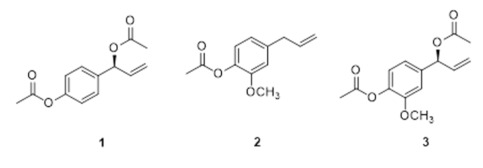				
Compound	Name	^a^ IC_50_ (µM) (95% CI)		
		SKOV3	A2780	HEK293
**1**	1′-acetoxychavicol acetate (ACA)	11.45 (9.26–3.63)	11.31 (10.81–11.80)	33.64 (31.26–36.03)
**2**	eugenyl acetate	>200	179.84 (162.17–197.51)	>200
**3**	1′-acetoxyeugenol acetate (AEA)	14.13 (10.83–17.44)	10.01 (8.74–11.28)	14.88 (10.56–19.20)
	cisplatin	21.13 (20.01–22.25)	19 (17.37–20.63)	6.56 (5.87–7.26)

^a^ Concentrations that show 50% cell viability inhibition. CI; confidence interval.

## Data Availability

Data are contained within the article.

## Supplementary Materials

The following are available online at https://www.mdpi.com/article/10.3390/antiox11020293/s1, Figure S1: TLC analysis of 1′-acetoxyeugenol acetate (AEA); Plate: Silica gel 60 F_254_ (Merck); Solvent system: *n*-hexane/EtOAc (65:35); A: UV 254 nm, B: day light after charring with 20% H_2_SO_4_; Figure S2: An ultra performance liquid chromatography (UPLC)-photodiode array (PDA) chromatogram of 1′-acetoxyeugenol acetate; Solvent system: (A) 0.1% formic acid in water (B) 0.1% formic acid in acetonitrile; Gradient: 0–0.5 min 10% B, 0.5–7.5 min 100% B, 7.5–9.5 min 100% B, 9.5-10.0 min 10% B; Figure S3: The ^1^H-nuclear magnetic resonance (500 MHz, CDCl_3_) spectrum of 1′-acetoxyeugenol acetate; Figure S4: The ^13^C- nuclear magnetic resonance (125 MHz, CDCl_3_) spectrum of 1′-acetoxyeugenol acetate; Figure S5: Effect of water extract on cell viability in human breast cancer MCF7 and human colon cancer CaCo2 cells; Figure S6: A schematic diagram summarizing the apoptosis-inducing effect of AEA in human ovarian cancer cells; Table S1: The primary antibodies used in Western blotting analysis; Table S2: Cytotoxic activity of ACA and AEA from the EtOH-extract of Thai ginger in human breast cancer MCF7 cells.

## References

[1] Siegel R.L., Miller K.D., Fuchs H.E., Jemal A. (2021). Cancer Statistics, 2021. CA Cancer J. Clin..

[2] Sung H., Ferlay J., Siegel R.L., Laversanne M., Soerjomataram I., Jemal A., Bray F. (2021). Global cancer statistics 2020: GLOBOCAN estimates of incidence and mortality worldwide for 36 cancers in 185 countries. CA Cancer J. Clin..

[3] Pokhriyal R., Hariprasad R., Kumar L., Hariprasad G. (2019). Chemotherapy Resistance in Advanced Ovarian Cancer Patients. Biomark. Cancer.

[4] Kaushik D., Yadav J., Kaushik P., Sacher D., Rani R. (2011). Current pharmacological and phytochemical studies of the plant Alpinia galanga. Zhong Xi Yi Jie He Xue Bao.

[5] Kanchanakunjara T., Chantachon S., Koseyayothin M., Kuljanabhagavad T. (2015). Traditional curry pastes during Sukhothai to Ratthanakosin: The subjective experience of the past and present. Asian Cult. Hist..

[6] Farnsworth N.R., Bunapraphatsara N. (1992). Thai Medicinal Plants Recommended for Primary Health Care System.

[7] Basri A.M., Taha H., Ahmad N. (2017). A review on the pharmacological activities and phytochemicals of alpinia officinarum (galangal) extracts derived from bioassay-guided fractionation and isolation. Pharmacogn. Rev..

[8] Matsuda H., Morikawa T., Managi H., Yoshikawa M. (2003). Antiallergic principles from Alpinia galanga: Structural requirements of phenylpropanoids for inhibition of degranulation and release of TNF-alpha and IL-4 in RBL-2H3 cells. Bioorg. Med. Chem. Lett..

[9] Beristain-Bauza S.D., Hernandez-Carranza P., Cid-Perez T.S., Avila-Sosa R., Ruiz-Lopez I.I., Ochoa-Velasco C.E. (2019). Antimicrobial Activity of Ginger (*Zingiber officinale*) and Its Application in Food Products. Food Rev. Int..

[10] Stoilova I., Krastanov A., Stoyanova A., Denev P., Gargova S. (2007). Antioxidant activity of a ginger extract (*Zingiber officinale*). Food Chem..

[11] Karuppiah P., Rajaram S. (2012). Antibacterial effect of Allium sativum cloves and *Zingiber officinale* rhizomes against multiple-drug resistant clinical pathogens. Asian Pac. J. Trop. Biomed..

[12] Habib S.H., Makpol S., Abdul Hamid N.A., Das S., Ngah W.Z., Yusof Y.A. (2008). Ginger extract (*Zingiber officinale*) has anti-cancer and anti-inflammatory effects on ethionine-induced hepatoma rats. Clinics.

[13] Morikawa T., Ando S., Matsuda H., Kataoka S., Muraoka O., Yoshikawa M. (2005). Inhibitors of nitric oxide production from the rhizomes of Alpinia galanga: Structures of new 8-9′ linked neolignans and sesquineolignan. Chem. Pharm. Bull..

[14] Awang K., Azmi M.N., Aun L.I., Aziz A.N., Ibrahim H., Nagoor N.H. (2010). The apoptotic effect of 1′s-1′-acetoxychavicol acetate from Alpinia conchigera on human cancer cells. Molecules.

[15] Baradwaj R.G., Rao M.V., Senthil Kumar T. (2017). Novel purification of 1′S-1′-Acetoxychavicol acetate from Alpinia galanga and its cytotoxic plus antiproliferative activity in colorectal adenocarcinoma cell line SW480. Biomed. Pharmacother..

[16] Matsuda H., Pongpiriyadacha Y., Morikawa T., Ochi M., Yoshikawa M. (2003). Gastroprotective effects of phenylpropanoids from the rhizomes of Alpinia galanga in rats: Structural requirements and mode of action. Eur. J. Pharmacol..

[17] Pang X., Zhang L., Lai L., Chen J., Wu Y., Yi Z., Zhang J., Qu W., Aggarwal B.B., Liu M. (2011). 1′-Acetoxychavicol acetate suppresses angiogenesis-mediated human prostate tumor growth by targeting VEGF-mediated Src-FAK-Rho GTPase-signaling pathway. Carcinogenesis.

[18] Batra V., Syed Z., Gill J.N., Coburn M.A., Adegboyega P., Di Giovanni J., Mathis J.M., Shi R., Clifford J.L., Kleiner-Hancock H.E. (2012). Effects of the tropical ginger compound,1'-acetoxychavicol acetate, against tumor promotion in K5.Stat3C transgenic mice. J. Exp. Clin. Cancer Res..

[19] Kawabata K., Tanaka T., Yamamoto T., Ushida J., Hara A., Murakami A., Koshimizu K., Ohigashi H., Stoner G.D., Mori H. (2000). Suppression of N-nitrosomethylbenzylamine-induced rat esophageal tumorigenesis by dietary feeding of 1′-acetoxychavicol acetate. Jpn. J. Cancer Res..

[20] Kobayashi Y., Nakae D., Akai H., Kishida H., Okajima E., Kitayama W., Denda A., Tsujiuchi T., Murakami A., Koshimizu K. (1998). Prevention by 1′-acetoxychavicol acetate of the induction but not growth of putative preneoplastic, glutathione S-transferase placental form-positive, focal lesions in the livers of rats fed a choline-deficient, L-amino acid-defined diet. Carcinogenesis.

[21] Tanaka T., Kawabata K., Kakumoto M., Makita H., Matsunaga K., Mori H., Satoh K., Hara A., Murakami A., Koshimizu K. (1997). Chemoprevention of azoxymethane-induced rat colon carcinogenesis by a xanthine oxidase inhibitor, 1′-acetoxychavicol acetate. Jpn. J. Cancer Res..

[22] Kojima-Yuasa A., Matsui-Yuasa I. (2020). Pharmacological Effects of 1′-Acetoxychavicol Acetate, a Major Constituent in the Rhizomes of Alpinia galanga and Alpinia conchigera. J. Med. Food.

[23] Pradubyat N., Giannoudis A., Elmetwali T., Mahalapbutr P., Palmieri C., Mitrpant C., Ketchart W. (2021). 1′-Acetoxychavicol Acetate from Alpinia galanga Represses Proliferation and Invasion, and Induces Apoptosis via HER2-signaling in Endocrine-Resistant Breast Cancer Cells. Planta Med..

[24] Noro T., Sekiya T., Katoh M., Oda Y., Miyase T., Kuroyanagi M., Ueno A., Fukushima S. (1988). Inhibitors of xanthine oxidase from Alpinia galanga. Chem. Pharm. Bull..

[25] Heo D.S., Park J.G., Hata K., Day R., Herberman R.B., Whiteside T.L. (1990). Evaluation of tetrazolium-based semiautomatic colorimetric assay for measurement of human antitumor cytotoxicity. Cancer Res..

[26] Li J., Yuan J. (2008). Caspases in apoptosis and beyond. Oncogene.

[27] Liou G.Y., Storz P. (2010). Reactive oxygen species in cancer. Free Radic. Res..

[28] Wada T., Penninger J.M. (2004). Mitogen-activated protein kinases in apoptosis regulation. Oncogene.

[29] Khodaie L., Sadeghpoor O. (2015). Ginger from ancient times to the new outlook. Jundishapur J. Nat. Pharm. Prod..

[30] Chouni A., Paul S. (2018). A review on phytochemical and pharmacological potential of Alpinia galanga. Pharmacogn. J..

[31] Matsuda H., Ando S., Morikawa T., Kataoka S., Yoshikawa M. (2005). Structure-activity relationships of 1′S-1′-acetoxychavicol acetate for inhibitory effect on NO production in lipopolysaccharide-activated mouse peritoneal macrophages. Bioorg. Med. Chem. Lett..

[32] Singh Y., Kalita J. (2012). Effects of Methanolic extract of Alpinia galanga from Manipur (India) on uterus of ovariectomised C3H albino mice. Int. Res. J. Pharm..

[33] Samarghandian S., Hadjzadeh M.A., Afshari J.T., Hosseini M. (2014). Antiproliferative activity and induction of apoptotic by ethanolic extract of Alpinia galanga rhizhome in human breast carcinoma cell line. BMC Complement. Altern. Med..

[34] Suja S., Chinnaswamy P. (2008). Inhibition of in vitro cytotoxic effect evoked by Alpinia galanga and Alpinia officinarum on PC-3 cell line. Anc. Sci. Life.

[35] Hadjzadeh M.A., Ghanbari H., Keshavarzi Z., Tavakol-Afshari J. (2014). The Effects of Aqueous Extract of Alpinia Galangal on Gastric Cancer Cells (AGS) and L929 Cells in Vitro. Iran. J. Cancer Prev..

[36] Muangnoi P., Lu M., Lee J., Thepouyporn A., Mirzayans R., Le X.C., Weinfeld M., Changbumrung S. (2007). Cytotoxicity, apoptosis and DNA damage induced by Alpinia galanga rhizome extract. Planta Med..

[37] Dixon R.A., Paiva N.L. (1995). Stress-Induced Phenylpropanoid Metabolism. Plant Cell.

[38] Ichikawa H., Takada Y., Murakami A., Aggarwal B.B. (2005). Identification of a novel blocker of I kappa B alpha kinase that enhances cellular apoptosis and inhibits cellular invasion through suppression of NF-kappa B-regulated gene products. J. Immunol..

[39] Ito K., Nakazato T., Murakami A., Ohigashi H., Ikeda Y., Kizaki M. (2005). 1′-Acetoxychavicol acetate induces apoptosis of myeloma cells via induction of TRAIL. Biochem. Biophys. Res. Commun..

[40] Phuah N.H., In L.L., Azmi M.N., Ibrahim H., Awang K., Nagoor N.H. (2013). Alterations of microRNA expression patterns in human cervical carcinoma cells (Ca Ski) toward 1′S-1′-acetoxychavicol acetate and cisplatin. Reprod. Sci..

[41] Phuah N.H., Azmi M.N., Awang K., Nagoor N.H. (2017). Down-Regulation of MicroRNA-210 Confers Sensitivity towards 1′S-1′-Acetoxychavicol Acetate (ACA) in Cervical Cancer Cells by Targeting SMAD4. Mol. Cells.

[42] Wang H., Shen L., Li X., Sun M. (2013). MicroRNAs contribute to the anticancer effect of 1′-acetoxychavicol acetate in human head and neck squamous cell carcinoma cell line HN4. Biosci. Biotechnol. Biochem..

[43] Liew S.K., Azmi M.N., In L., Awang K., Nagoor N.H. (2017). Anti-proliferative, apoptotic induction, and anti-migration effects of hemi-synthetic 1'S-1'-acetoxychavicol acetate analogs on MDA-MB-231 breast cancer cells. Drug Des. Dev. Ther..

[44] In L.L., Azmi M.N., Ibrahim H., Awang K., Nagoor N.H. (2011). 1′S-1′-acetoxyeugenol acetate: A novel phenylpropanoid from Alpinia conchigera enhances the apoptotic effects of paclitaxel in MCF-7 cells through NF-kappaB inactivation. Anti-Cancer Drugs.

[45] Jayasingh Chellammal H.S., Veerachamy A., Ramachandran D., Gummadi S.B., Manan M.M., Yellu N.R. (2019). Neuroprotective effects of 1′delta-1′-acetoxyeugenol acetate on Abeta(25–35) induced cognitive dysfunction in mice. Biomed. Pharmacother..

[46] Angulo P., Kaushik G., Subramaniam D., Dandawate P., Neville K., Chastain K., Anant S. (2017). Natural compounds targeting major cell signaling pathways: A novel paradigm for osteosarcoma therapy. J. Hematol. Oncol..

[47] Sarkar F.H., Li Y., Wang Z., Kong D. (2009). Cellular signaling perturbation by natural products. Cell Signal..

[48] Elmore S. (2007). Apoptosis: A review of programmed cell death. Toxicol. Pathol..

[49] Kasibhatla S., Tseng B. (2003). Why target apoptosis in cancer treatment?. AACR J..

[50] Tummers B., Green D.R. (2017). Caspase-8: Regulating life and death. Immunol. Rev..

[51] Millimouno F.M., Dong J., Yang L., Li J., Li X. (2014). Targeting apoptosis pathways in cancer and perspectives with natural compounds from mother nature. Cancer Prev. Res..

[52] Kato R., Matsui-Yuasa I., Azuma H., Kojima-Yuasa A. (2014). The synergistic effect of 1′-acetoxychavicol acetate and sodium butyrate on the death of human hepatocellular carcinoma cells. Chem. Biol. Interact..

[53] Zorov D.B., Juhaszova M., Sollott S.J. (2006). Mitochondrial ROS-induced ROS release: An update and review. Biochim. Biophys. Acta.

[54] Di Meo S., Reed T.T., Venditti P., Victor V.M. (2016). Role of ROS and RNS Sources in Physiological and Pathological Conditions. Oxidative Med. Cell. Longev..

[55] Juhasz A., Ge Y., Markel S., Chiu A., Matsumoto L., van Balgooy J., Roy K., Doroshow J.H. (2009). Expression of NADPH oxidase homologues and accessory genes in human cancer cell lines, tumours and adjacent normal tissues. Free. Radic. Res..

[56] Kim S.M., Hwang K.A., Choi K.C. (2018). Potential roles of reactive oxygen species derived from chemical substances involved in cancer development in the female reproductive system. BMB Rep..

[57] Pearson G., Robinson F., Beers Gibson T., Xu B.E., Karandikar M., Berman K., Cobb M.H. (2001). Mitogen-activated protein (MAP) kinase pathways: Regulation and physiological functions. Endocr. Rev..

