# Supplementary material for: 1′-Acetoxyeugenol Acetate Isolated from Thai Ginger Induces Apoptosis in Human Ovarian Cancer Cells by ROS Production via NADPH Oxidase
Source: Antioxidants (Basel). 2022 Jan 31;11(2):293. doi: 10.3390/antiox11020293 (PMC8868116; doi:10.3390/antiox11020293)
Supplement: Supplementary file 1 [file antioxidants-11-00293-s001.zip › antioxidants-1556978-supplementary.pdf]

Supporting Information

1′-Acetoxyeugenol acetate isolated from Thai ginger induces apoptosis in human ovarian cancer cells by ROS production via NADPH oxidase

Ju-Yeon Choi[1], Na-Kyung Lee[1], Yi-Yue Wang[1], Joon-Pyo Hong[1], So-Ri Son[1], Da-Hye Gu[2], Dae Sik Jang[1,2], Jung-Hye Choi[1,2]

Affiliation

[1] Department of Biomedical and Pharmaceutical Sciences, Kyung Hee University, Seoul, South Korea

[2] College of Pharmacy, Kyung Hee University, Seoul, South Korea

Correspondence

Prof. Jung-Hye Choi

College of Pharmacy, Kyung Hee University, 26 Kyungheedae-ro, Dongdaemoon-gu, Seoul 02447, South Korea

Phone: +8229612246. Fax: +8229620860

jchoi@khu.ac.kr

Contents

Figure S1. TLC analysis of 1′-acetoxyeugenol acetate (AEA); Plate: Silica gel 60 F_{254} (Merck); Solvent system: *n*-hexane/EtOAc (65:35); **A**: UV 254nm, **B**: day light after charring with 20 % H_2SO_4.

Figure S2. An ultra performance liquid chromatography (UPLC)- photodiode array (PDA) chromatogram of 1′-acetoxyeugenol acetate; Solvent system: (A) 0.1% formic acid in water (B) 0.1% formic acid in acetonitrile; Gradient: 0–0.5 min 10% B, 0.5–7.5 min 100% B, 7.5–9.5 min 100% B, 9.5-10.0 min 10% B.

Figure S3. The ^{1}H-nuclear magnetic resonance (500 MHz, $CDCl_3$) spectrum of 1′-acetoxyeugenol acetate.

Figure S4. The ^{13}C- nuclear magnetic resonance (125 MHz, $CDCl_3$) spectrum of 1′-acetoxyeugenol acetate.

Figure S5. Effect of water extract on cell viability in human breast cancer MCF7 and human colon cancer CaCo2 cells.

Figure S6. A schematic diagram summarizing the apoptosis-inducing effect of AEA in human ovarian cancer cells.

Table S1. The primary antibodies used in Western blotting analysis.

Table S2. Cytotoxic activity of ACA and AEA from the EtOH-extract of Thai ginger in human breast cancer MCF7 cells.

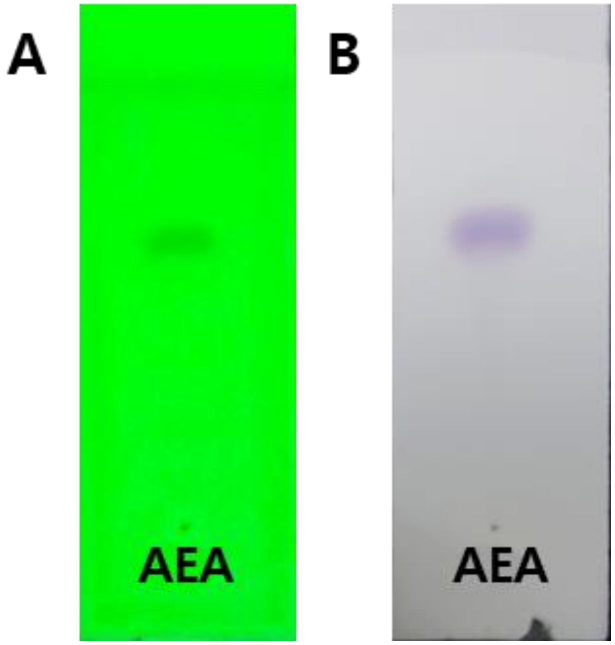

Figure S1. TLC analysis of 1′-acetoxyeugenol acetate (AEA); Plate: Silica gel 60 F_{254} (Merck); Solvent system: *n*-hexane/EtOAc (65:35); **A**: UV 254nm, **B**: day light after charring with 20 % H_2SO_4.

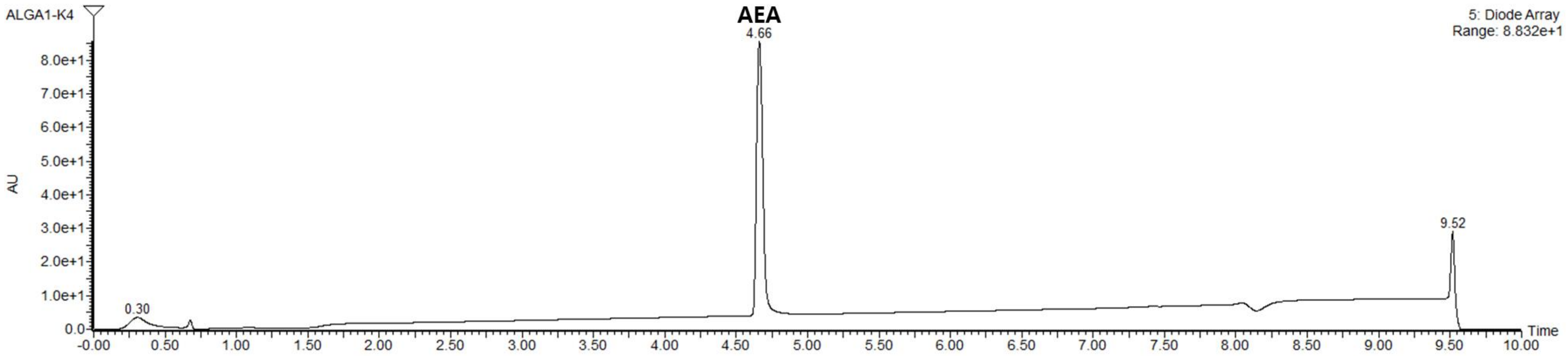

Figure S2. An ultra performance liquid chromatography **(**UPLC)- photodiode array (PDA) chromatogram of 1′-acetoxyeugenol acetate; Solvent system: (A) 0.1% formic acid in water (B) 0.1% formic acid in acetonitrile; Gradient: 0–0.5 min 10% B, 0.5–7.5 min 100% B, 7.5–9.5 min 100% B, 9.5-10.0 min 10% B.

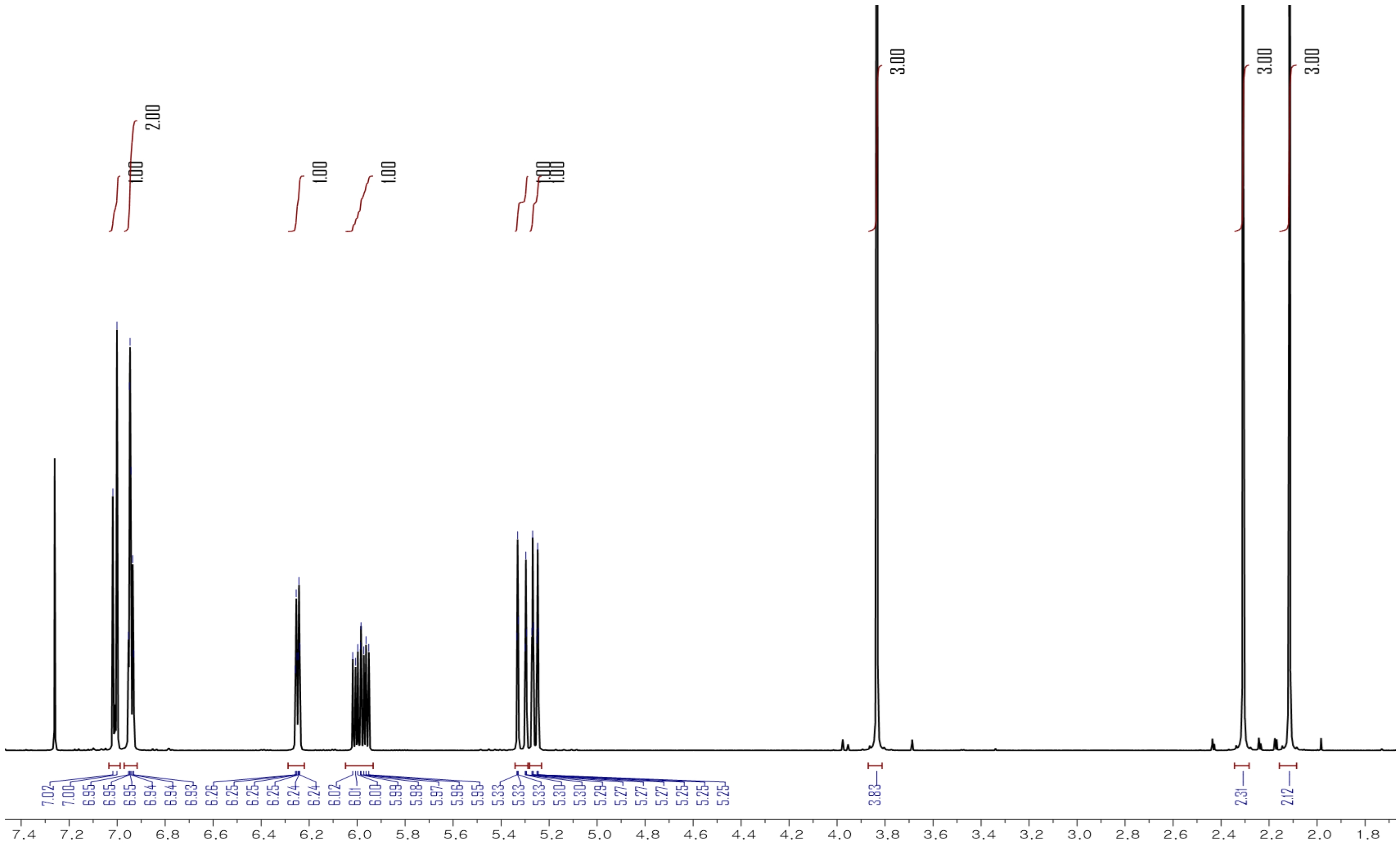

Figure S3. The ^{1}H-nuclear magnetic resonance (500 MHz, $CDCl_3$) spectrum of 1′-acetoxyeugenol acetate

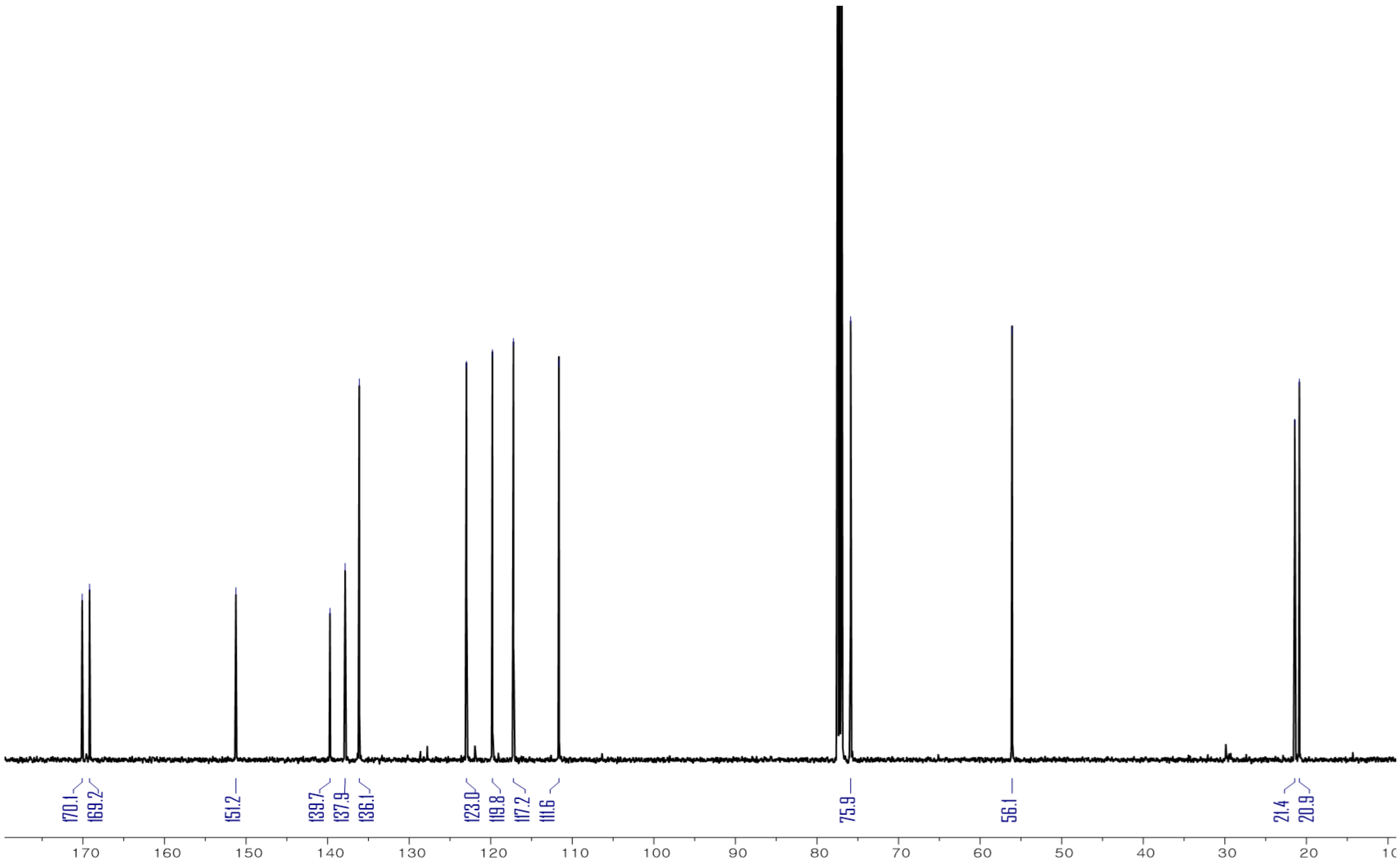

Figure S4. The ^{13}C- nuclear magnetic resonance (125 MHz, $CDCl_3$) spectrum of 1′-acetoxyeugenol acetate

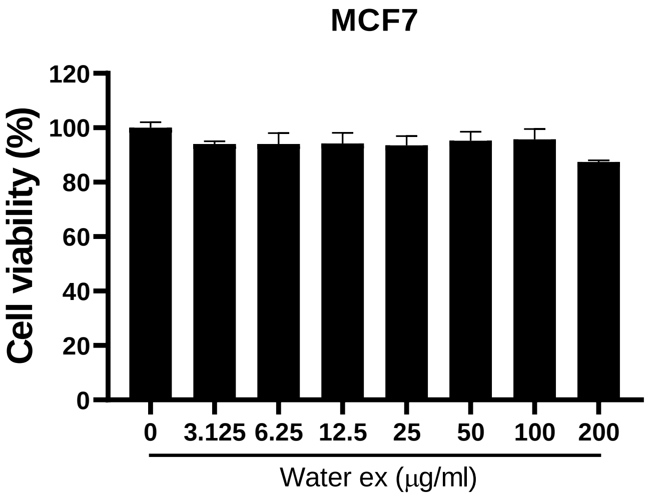

Figure S5. Effect of water extract on cell viability in human breast cancer MCF7 and human colon cancer CaCo2 cells. MCF7 and CaCo2 cells were treated with the indicated concentration of water extract for 48 h. MTT assay was performed for the cell viability $*p < 0.05$ compared with control

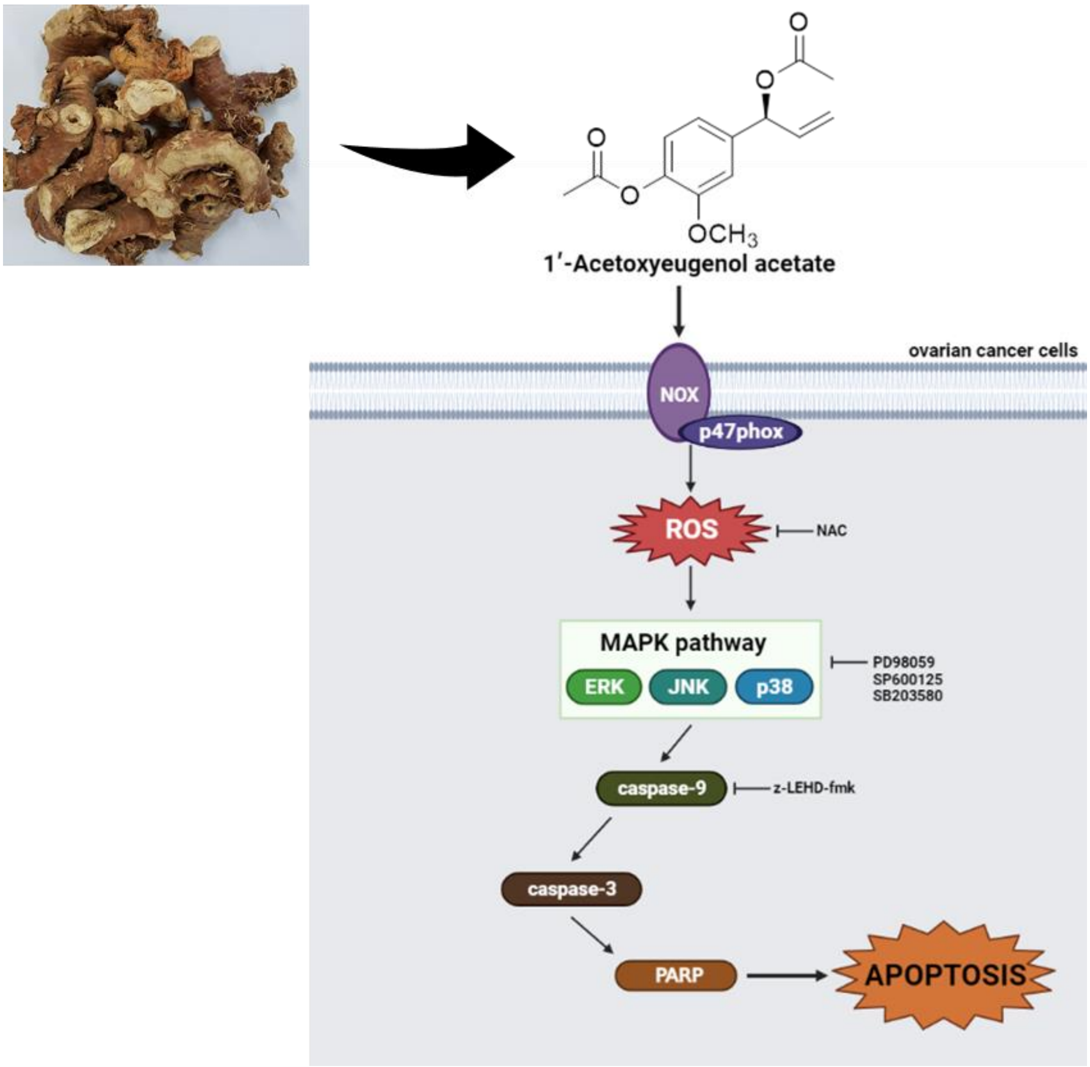

Figure S6. A schematic diagram summarizing the apoptosis-inducing effect of AEA in human ovarian cancer cells

Table S1. The primary antibodies used in Western blotting analysis

A list of antibodies used for western blot			
Antibodies name	Concentration	Species	Company
Caspase-9	1:1000	Rabbit	Cell Signaling Technology
Caspase-3	1:1000	Rabbit	Cell Signaling Technology
Caspase-8	1:1000	Rabbit	Santa Cruz Biotechnology
total ERK1/2	1:1000	Mouse	Santa Cruz Biotechnology
total p38	1:1000	Mouse	Santa Cruz Biotechnology
total JNK	1:1000	Mouse	Santa Cruz Biotechnology
phosphor-ERK1/2	1:1000	Mouse	Santa Cruz Biotechnology
phosphor-JNK	1:1000	Mouse	Santa Cruz Biotechnology
phosphor-p38	1:1000	Mouse	Santa Cruz Biotechnology
β-actin	1:1000	Mouse	Santa Cruz Biotechnology

Table S2. Cytotoxic activity of ACA and AEA from the EtOH-extract of Thai ginger in human breast cancer MCF7 cells

**Name**	[a] **IC_{50}** (μM) **(95% CI)**
1'-acetoxychavicol acetate (ACA)	19.50 (19.07-19.93)
1'-acetoxyeugenol acetate (AEA)	29.63 (20.76-38.51)

Notes: [a] IC_{50} is defined as the concentration that results in a 50% decrease in the number of cells compared to that of the control groups. CI; confidence interval
